# Molecular docking of phenolic compounds and screening of antioxidant and antidiabetic potential of *Moringa oleifera* ethanolic leaves extract from Qassim region, Saudi Arabia

**DOI:** 10.1016/j.sjbs.2021.10.021

**Published:** 2021-10-13

**Authors:** Sridevi Chigurupati, Atheer Al-murikhy, Suliman A Almahmoud, Yosif Almoshari, Amira Saber Ahmed, Shantini Vijayabalan, Shatha Ghazi Felemban, Vasanth Raj Palanimuthu

**Affiliations:** a Department of Medicinal Chemistry and Pharmacognosy, College of Pharmacy, Qassim University, Buraidah 52571, Saudi Arabia; b Department of Pharmaceutics, College of Pharmacy, Jazan University, Jazan 45142, Saudi Arabia; c Hormones Department, Medical Research and Clinical Studies Institute, National Research Centre, Giza, Egypt.; d School of Pharmacy, Faculty of Health and Medical Sciences, Taylor's University, Subang Jaya, Kuala Lumpur 47500, Malaysia; e Department of Medical Laboratory Science, Fakeeh College for Medical Sciences, Jeddah, Kingdom of Saudi Arabia; f Department of Pharmaceutical Biotechnology, JSS College of Pharmacy, JSS Academy of Higher Education & Research, Ooty, Nilgiris, Tamilnadu, India

**Keywords:** Moringa oleifera, Phytochemical, Physicochemical, Antioxidant, Antidiabetic, Molecular docking

## Abstract

**Introduction:**

Oxidative stress is crucial in diabetic pathophysiology, hence the prerequisite of ingesting naturally derived antioxidants as a remedial target. This study investigates the naturally occurring antioxidant and antidiabetic potential of Moringa oleifera ethanolic leaves extract.

**Methods:**

Moringa oleifera leaves were macerated (MOLE) by using 70% ethanol. Physiochemical and phytochemical examinations of MOLE was assayed using standard methods. The antioxidant activity was analyzed by DPPH (1, 1-diphenyl-2-picrylhydrazil) radical scavenging assay. In vitro antidiabetic was analyzed by pancreatic α-amylase enzyme inhibitory assay. The molecular docking was performed using AutoDock Vina v1.1.2 in PyRx 30.8.

**Results:**

Ethanolic extraction of MOLE by maceration technique, 14 % yield. Loss on drying, foreign organic matters and total ash value of OLE showed 0.27 w/w, 0.8 % and 19 %, respectively. Phytochemical test on MOLE confirmed starch, carbohydrate, flavonoid, gum, glycoside, saponin, tannin, and phenol presences. The total phenolic and flavonoid contents of MOLE are 260 mg GAE/g and 755 mg RUE/g of extract. MOLE (IC 50 55.6 ± 0.18 µg/mL) showed functional DPPH scavenging assay comparable to ascorbic acid (IC 50 46.71 ± 0.24 µg/mL). In the alpha-amylase inhibitory activity, Acarbose showed an IC 50 value of 19.45 ± 0.26 µg/mL, while MOLE portrayed an IC 50 value of 27.54 ± 0.07 µg/mL. Docking studies revealed that most phenolic compounds found within MOLE have minimum docking scores and high binding affinity against Human pancreatic alpha-amylase.

**Conclusions:**

The invitro and docking results suggest that MOLE has been a viable natural bioactive source and might be a great potential source for future antidiabetic medicine.

## 1 Introduction

Oxidative stress has caused diseases such as hyperlipidaemia, hypertension and malignancies. Also, oxidative stress is harmful as oxygen free radicals damage biological molecules such as lipids, proteins and DNA. Some of the markers used in detecting oxidative stress are ubiquinol-10, isoprostan and lipid hydroperoxides. Human bodies synthesise antioxidants naturally as catalase to inhibit free radical damages through neutralisation (Phaniendra et al., 2015).

Diabetes mellitus (DM) is a slow damaging disease known worldwide due to the low insulin production or created damaged insulin that is unusable for body cells. Three types of diabetes are labelled as gestational diabetes, type-1 and type-2 diabetes. Type-1 diabetes mellitus causes autoimmune damages responsible for insulin production, especially beta cells in the pancreas. Type-2 diabetes mellitus leads to inefficient insulin production. Gestational diabetes occurs in pregnant women with no diabetic history, where high blood sugar level happens throughout pregnancy (Siddiqui et al., 2013). Drugs sourced from medicinal plants help to control diabetic progression in traditional settings. These traditional medicinal plants are preferable to synthetic ones for less toxicity and side effects (Yuan et al., 2016). Degradation of the dietary starch proceeds rapidly and leads to elevated PPHG (postprandial hyperglycemia). It has been shown that activity of HPA (human pancreatic α-amylase) in the small intestine correlates to an increase in postprandial glucose levels, the control of which is therefore an important aspect in treatment of type-2 diabetes (Khajaria et al., 2013) Inhibitors of pancreatic α-amylase delay carbohydrate digestion causing a reduction in the rate of glucose absorption and lowering the postprandial serum glucose levels. In 1970 s, it was realized that inhibition of all or some of the intestinal disaccharidases and pancreatic α-amylase by inhibitors could regulate the absorption of carbohydrate and these inhibitors could be used therapeutically in the oral treatment of the noninsulin-dependent diabetes mellitus ie., type-2 diabetes (Vijan et al.,1997).

There is an increase in natural product usage towards fulfilling its role of treating and preventing diseases in humans. Therefore, drugs with natural sources have higher efficacy and are a drug of choice than synthetic ones (Kadir et al., 2013). *Moringa oleifera*, commonly known as horseradish tree, is a pan-tropical species of large-sized trees sourced from the sub-Himalayan regions in North India, Bangladesh, Pakistan and Afghanistan. Other common names are moringa, benzoil tree, drumstick tree or horseradish (Fahey, 2005). Most parts of *M. oleifera*, namely seeds, leaves and pods, were used as components in traditional medicine (Abdull Razis et al., 2014, Gopalakrishnan et al., 2016). The leaves and pods were commonly consumed in India and South Africa (Ahmad et al., 2018). Then, parts of *M. oleifera* are examined for medicinal properties associated with different bioactive ingredients, such as phenolic acids, vitamins, isothiocyanates, flavonoids, saponins, and tannins that exist as essential quantities of ingredients in a plant. Leaves of *M. oleifera* are used in different types of chronic diseases, including dyslipidemia, hypertension, diabetes mellitus, fatty liver, malignancy, pain and fever reduction, asthma, and inflammation (Martín et al., 2013).

Based on Sutalangka et al. (2013), leaves extract of horseradish tree helps to enhance spatial memory and reduced neurodegeneration in the hippocampus of ICV-induced AF64A dementia Wistar rats. It has reduced malondialdehyde and acetylcholinesterase, enhanced superoxide dismutase and catalase parameters (Sutalangka et al., 2013). Methanolic extract in *M. oleifera (L)* helps in decreasing atherogenic index, serum cholesterol, low-density lipoproteins (LDL), triacylglyceride, and very-low-density lipoprotein (VLDL). The aspects of the methanolic extract help to increase high-density lipoproteins (HDL) in high fat-induced hyperlipidemia on albino Wistar rats (Jain et al., 2010). Leaves, seeds and bark extracts in *M. oleifera* were reported as containing anti-cancer activities towards breast and colorectal cancer cell lines (Al-Asmari et al., 2015).

Hence, these plants can inhibit alpha-amylase is further studies to develop a cure for DM. In the present study, we targeted *Moringa oleifera* leaves to find the flavonoid and phenolic contents and then investigate for antidiabetic and antioxidant potentials.

## 2 Materials and methods

### 2.1 Reagents

Acarbose and pancreatic α-amylase, and Folin–Ciocâlteu reagent were obtained from Sigma- Aldrich Corporations, USA. Ethanol, gallic acid, sodium carbonate and sodium phosphate buffer were purchased from Fouz Chemical Company, Saudi. DNS (3, 5-Dinitrosalicylic acid) and phosphate buffer were attained from Merck Millipore Corporation, USA. DPPH (2,2-diphenyl-1-picrylhydrazyl) was obtained from Cayman Chemical Company, USA. The remaining chemicals or reagents were of analytical grade.

### 2.2 Collection of plant material and leaves extraction

In February 2019, the leaves from *M. oleifera* were collected from Alosimi farms in the Qassim region, Kingdom of Saudi Arabia, GPS location (24°27′00.4″N 46°08′33.5″E). The plant authentication was affirmed by the Department of Pharmacognosy, Qassim University, Saudi Arabia (Ref. No: QA/FOP/07). Grounded dried leaves (50 g) added with of70 % ethanol (200 mL) and macerated for five days (cold maceration at room temperature). Methodical extraction was done from the residual plant material, and repeated processes were done till colourless supernatant liquid was obtained. Then, the extract solution was filtered with a muslin cloth, is subjected to rotary evaporation, and this *M. oleifera* leaves extract (MOLE) was freeze-dried. The percentage yield of the MOLE extract was calculated (Chigurupati et al., 2018).

### 2.3 Phytochemical and physicochemical assessments

The phytochemical assessment was studied on MOLE based on standard methods. These assessments were tested on the extract towards distinguishing the presence of constituents such as starch, carbohydrate, phenol, protein, glycoside, gum, flavonoid, tannin and saponin (Chigurupati et al., 2017, Majid et al., 2015, Roopashree et al., 2008).

Physiochemical assessments were studied on MOLE as standard methods. Ash value of MOLE was measured from the calculation of residual content after igniting around 650–700 °C (Roy et al., 2013). The percentage of total ash and foreign organic material was calculated. The moisture content determines the loss post drying and is measured in grams.

### 2.4 Evaluation of total phenolic content

The preparation of the reaction mixture was done from 0.5 mL of ethanolic solution (0.1 mg/mL) extract, 2.5 mL of 0.75 % sodium bicarbonate solution, and 2.5 mL of 1 % Folin–Ciocalteu's reagent. The standard drug was prepared using Gallic acid of different concentrations (0.1–1.0 mg/mL) in ethanol. The samples and standards were kept at 37 °C for 30 min. The absorbance was taken at 765 nm using a UV/Vis spectrophotometer. These samples were prepared as triplicates, and the mean of absorbance was obtained. Absorbance data were represented as mean ± standard deviation (SD). A blank solution was prepared concomitantly with ethanol instead of extract solution. The calibration line was constructed from Gallic acid observations. TPC of MOLE was calculated and expressed as Gallic acid in GAE mg/g (Chigurupati et al., 2016a). The total phenol content (TPC) was calculated using Eq (1):

(1) TPC=(C×V)/m

C = Concentration of GA from the calibration curve (μg/mL), V = Extract volume (mL), m = extract weight (g)

### 2.5 Evaluation of total flavonoid content

The modified spectrophotometric method was used to evaluate the total flavonoid content (TFC) of MOLE. MOLE (0.2 mg/mL) was prepared with the use of methanol as a solvent. Rutin was used as a standard drug and prepared in different concentrations (10–1000 µg/mL) of methanol solvent. The reaction mixture was prepared from 3 mL of MOLE and 3 mL of 2 % aluminium chloride solution and dissolved in methanol. Then, it was incubated at room temperature (37 °C) for an hour. Absorbance was measured at 415 nm using a UV/Vis spectrophotometer. These samples were prepared as triplicate, and the mean of absorbance was obtained. These data were represented as mean ± SD. These processes were repeated, and a calibration line was constructed. TFC was calculated and expressed mg Rutin equivalents per gram of dry weight (RUE mg/g) (Chigurupati et al., 2017). The TFC was calculated using Eq. (2):

(2) TFC=(C×V)/m

C = Concentration of Rutin from the calibration curve (μg/mL), V = Extract volume (mL), m = extract weight (g)

### 2.6 DPPH radical scavenging assay

DPPH solution was formulated using Molyeux and Blois technique with slight modifications for antioxidant assay. The extract and standard ascorbic acid were done in different concentrations using absolute ethanol (10–1000 µg/mL). DPPH solution (500 µL) was added to the sample (500 µL) and incubated at room temperature in dark condition for approximate 20 min. Accordingly, the absorbance was measured at 517 nm (Chigurupati et al., 2016b). The percentage (%) of the free radical scavenging inhibitory assay was computed using Eq. (3):

(3) %Inhibition=(absorbance_control_–absorbance_sample_)/absorbance_control_x100

### 2.7 Alpha-Amylase enzymatic inhibitory assay

Acarbose (standard) and sample extract were prepared in different concentration (10–1,000 µg/mL). These samples (500 µL) are added to 0.5 mg/mL alpha-amylase solution (500 µL) that was prepared in 0.2 mM phosphate buffer (pH 6.9) and incubated at 25 °C for 10 min. 1 % Starch solution (500 µL) was prepared in a 0.02 M sodium phosphate buffer added, and incubated for 10 min at 25 °C. DNS (1 mL) is added and boiled for 5 min. These tubes were cooled at room temperature. Distilled water (10 mL) was added. The absorbance rate was measured at 540 nm (Noreen et al., 2017; Khazaria et al., 2013). The sample enzymatic inhibitory activity for the antidiabetic assay was computed as followed: using Eq. (3).

### 2.8 Molecular docking

The molecular docking of α-amylase has been done by downloading the X-ray crystal structure of the human pancreatic α-amylase complexing with mini-montbretin A (PDBID: 5E0F) (Williams et al., 2015). The ligand, and water atoms were removed, while the nonpolar hydrogens were merged. Then, the protein was minimized and optimized through AutoDock Tool (ADT), bundled with the MGLTools package (version 1.5.6) to add charges, polar hydrogen atoms, and set up rotatable bonds (Morris et al., 2009). The molecular docking was performed using AutoDock Vina v1.1.2 in PyRx 30.8 (Dallakyan et al., 2015). The active binding site of the α-amylase with the mini-montbretin A was chosen as the grid centres. The centre grid box dimensions were chosen to include all atoms of the ligand set. The site of the grid box in α-amylase was set at −7.946, 10.438, and −21.863 Å (for ×, y and z) by means of a grid of 40, 40, and 40 points (for ×, y and z). The structures of phenolic compounds found within Moringa oleifera L. extracts were retrieved from PubChem database (Kim et al., 2019). The phenolic compounds were minimized and optimized by using AutoDock Tool (ADT). The docking scores were resulted in the generated .log files. The output docking scores were defined as affinity binding (Kcal/mol). The ligands protein interactions were created by using the Discovery Studio version v19.1.0.18287 (BIOVIA, San Diego, CA, USA) (Dassault Systèmes BIOVIA, 2017).

## 3 Results

### 3.1 Phytochemical and physicochemical analysis

Present studies explore different types of phytoconstituents that demonstrate antioxidant and antidiabetic properties of *M. oleifera* leaves. Ethanolic extraction of *M. oleifera* by maceration is convenient, cost-effective, and produces more yield (14 %). Based on the summarised Table 1 (Nizioł-Łukaszewska et al., 2020) and the current study (Table 2), MOLE phytochemical screening showed the presence of saponin, flavonoid, gum, glycoside, tannin, phenol, starch, and carbohydrate reduction in plant extract activity due to the presence of phytoconstituents. Total ash value was used to distinguish the quality or purity of crude extract in powder form. This eliminates all organic traces in ashing vegetable drugs. After burning, ash contents in a crude extract resulted in the excess residue of naturally inorganic salt. Total ash value is 19 %, and foreign organic matter in MOLE is 0.8 %. Loss on drying (Gravimetric method) showed that moisture content is 0.27 w/w.

**Table 1:** Summary of phenolic compound reported found within Moringa oleifera L. Extracts (Nizioł-Łukaszewska et al., 2020).

Molecular formula	Molar mass (Da)	Identification
C_7_H_12_O_6_	192.2	Quinic acid
C_9_H_8_O_4_	180.2	Caffeic acid
C_16_H_18_O_9_	354.3	Chlorogenic acid
C_7_H_6_O_5_	170.1	Gallic acid
C_16_H_18_O_8_	339.0	Coumaroylquinic acid
C_21_H_20_O_11_	448.3	Astragalin
C_27_H_30_O_15_	594.5	Kaempferol-3-O-rutinoside
C_21_H_20_O_10_	432.1	Vitexin
C_27_H_30_O_16_	610.5	Rutin
C_23_H_22_O_13_	506.4	Quercetin-acetyl-glucoside
C_24_H_22_O_15_	550.4	Quercetin-malonyl-glucoside
C_21_H_20_O_12_	464.1	Isoquercetin
C_23_H_22_O_12_	490.4	Kaempferol acetyl glycoside
C_15_H_10_O_7_	302.2	Quercetin

**Table 2:** Phytochemical analysis of MOLE.

Phytochemical Constituents	M. oleifera
Saponin	+
Flavonoid	+
Gum	+
Tannin (Iron III)	+
Glycoside	+
Protein	–
Phenol	+
Carbohydrate	+
Starch	+

### 3.2 Total phenol and flavonoid contents

**Fig. 1:**
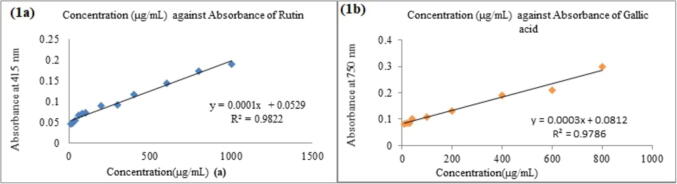
. (a). Standard curve of Rutin to estimate total flavonoid content; (b). Standard curve of Gallic acid to estimate total phenolic content.

As depicted in Fig. 1(a), the flavonoid content was denoted in Rutin equivalent (the standard curve equation: y = 0.0001 + 0.0529, r^2^ = 0.9822); the macerated ethanolic extract *M. oleifera* exhibited the flavonoid content of 755 mg RUE/g. As depicted in Fig. 1(b), the phenolic content was estimated using Folin-Ciocalteu reagent in terms of Gallic acid equivalent (standard curve equation: y = 0.0003x + 0.0812, r^2^ = 0.9786). The macerated ethanolic extract of *M. oleifera* has total phenolic content of 260 mg GAE/g.

### 3.3 Antioxidant assay

**Fig. 2:**
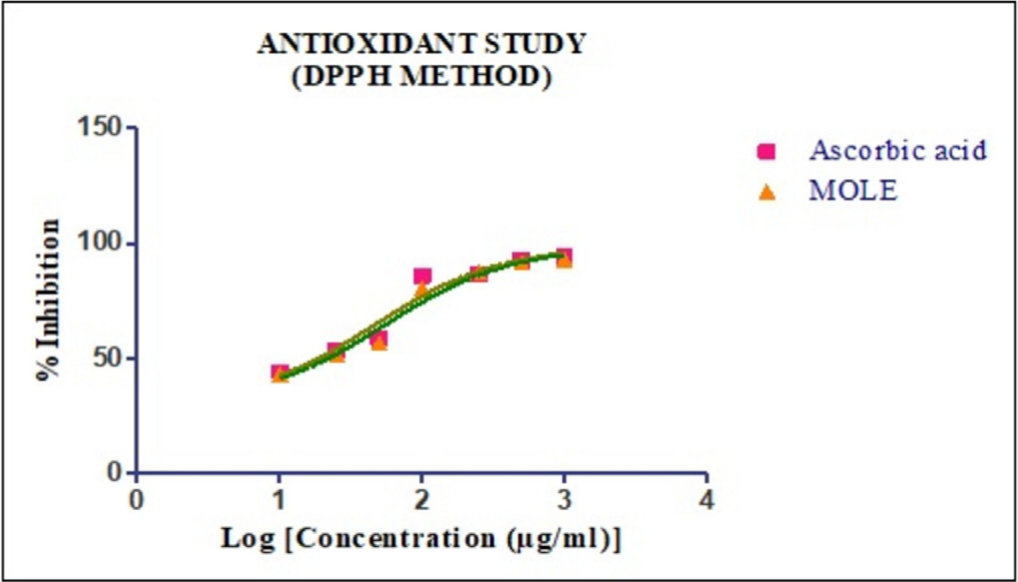
DPPH radical scavenging effects of MOLE and standard, Ascorbic acid.

The antioxidant assay is studied on MOLE and used the DPPH method. In the DPPH assay, the reagent was reduced from receiving hydrogen atoms or donating an electron and changed colours from violet to colourless or pale yellow (Sahoo et al., 2013). Ascorbic acid and samples were prepared in different concentrations ranging from 10 − 1000 µg/mL. As depicted in Fig. 2, MOLE (IC_50_ ± SEM: 55.6 ± 0.18 µg/mL) portrayed comparable to the ascorbic acid (IC_50_ ± SEM: 46.71 ± 0.24 µg/mL).

### 3.4 In vitro antidiabetic assay

As depicted in Table 3, the standard Acarbose and MOLE were examined at various concentrations. In the alpha-amylase inhibitory activity, Acarbose and MOLE showed enzymatic inhibition with an IC_50_ value of 19.45 ± 0.26 µg/mL and IC_50_ value of 27.54 ± 0.07 µg/mL, respectively.

**Table 3:** Alpha-Amylase (%) inhibition of MOLE.

Concentration (µg/mL)	% inhibition of alpha-amylase	
	Acarbose	MOLE
10	31.67	25.00
25	41.67	41.67
50	75.00	62.50
100	83.33	73.33
250	88.33	85.00
500	90.83	88.33
1000	93.33	91.67
IC_50_ ± SEM	19.45 ± 0.26^a^	27.54 ± 0.07^a^

Note ^a^ SEM using Graph Pad prism 5 (n = 3).

### 3.5 The molecular docking

Most of phenolic compounds found within MOLE showed good binding affinity against HPA (Table 4). The flavonol di-hexose such as rutin and nicotiflorin (Kaempferol-3-O-rutinoside) showed the most potent binding affinity against HPA than flavonol hexose. The docking scores for rutin and nicotiflorin are − 9.40 kcal/mol, and − 9.10 kcal/mol, respectively. In contrast, the docking scores for flavone mono-hexose such as isoquercetin and astragalin are −8.80 kcal/mol, and − 8.50 kcal/mol, respectively. The quercetin acetyl-glucoside, quercetin malonyl-glucoside, and kaempferol acetyl-glycoside showed potent binding energy values against HPA, the docking scores for are − 9.20 kcal/mol, − 9.10 kcal/mol, and − 9.00 kcal/mol respectively. Quercetin analogues showed more minimum binding energy than kaempferol analogues. The flavone hexose showed lower binding affinty against HPA than flavonol hexose. On the other side, coumaroylquinic acid and chlorogenic acid are phenolic acid compounds and they showed favourably docking scores (Table 4). Consequently, these docking scores exposed that most phenolic compounds found within MOLE have minimum docking scores and high binding affinity against HPA, and thus they would likely bind and inhibit the HPA.

**Table 4:** The docking scores and the interacting residues of the phenolic compounds found within Moringa oleifera L. extracts against the human pancreatic α-amylase (HPA).

Comp.	Docking Scores (kcal/mol)	Interacting Residues
Acarbose	− 8.10	K200, E233, D300, H305
Quinic acid	− 6.60	E233, D300
Caffeic acid	− 6.70	W59, D197, E233, D300
Chlorogenic acid	− 8.00	W59, D197, E233, D300, H305
Gallic acid	− 6.00	D197, E233, D300
Coumaroylquinic acid	− 8.20	W59, D300, H305
Astragalin	− 8.50	E233, D300, H305
Kaempferol-3-O-rutinoside	− 9.10	W56, Q63, D197, E233, H305, D356
Vitexin	− 8.50	W56, H305, D356
Rutin	− 9.40	E233, H305, D356
Quercetin-acetyl-glucoside	− 9.20	W56, D300, H305, D356
Quercetin-malonyl-glucoside	− 9.10	D197, E233, H305
Isoquercetin	− 8.80	H305, D356
Kaempferol acetyl glycoside	− 8.10	D197, D300
Quercetin	− 8.10	W56, H305, D356

**Fig. 3:**
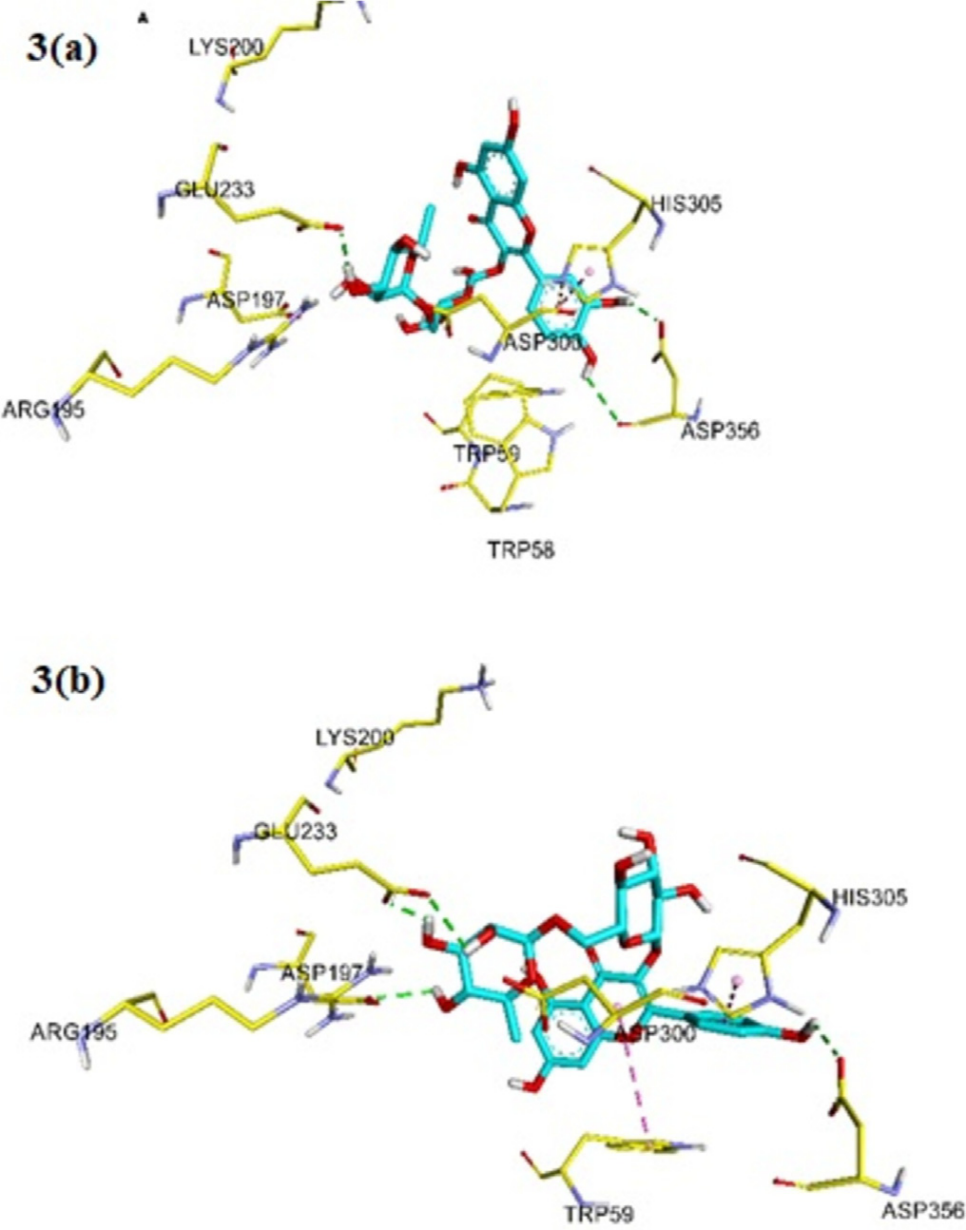
Ligands interactions between (a) Rutin against HPA; (b) Kaempferol-3-rutinoside against HPA: The H-bond interactions are shown as green dotted lines, and the π–π stacking interaction are shown as lavender dotted lines.

In addition, we studied the binding interactions of phenolic compounds found within MOLE against HPA to identify their inhibitory mechanism. The surface of the binding site of HPA protein is mostly surrounded by hydrophilic residues: W59, Q63, H101, Y151, R195, D197, K200, H201, E233, E240, I253, N298, D300, H305, and D356 (Williams et al., 2015). The binding interactions of phenolic compounds found within MOLE versus acarbose had sharing interactions. They have similar hydrogen bonding with E233 and D300. Besides, the aromatic rings of flavonoids and phenolic acids generate cation – π with His 305 and π - π stacking with W56 (Fig. 3). while the hydroxyl groups of flavonoids and phenolic acids form H-bonding with D197, E233m and D356 (Fig. 3). Therefore, the majority of phenolic compounds found within MOLE interact with the active site of HPA through the hydrogen bonding with D197, E233, D300, and D356, and hydrophobic interaction with W59, and H305.

## 4 Discussion

Moringa oleifera, a plant commonly found in many Asian and South-East Asian countries, contains numerous compounds with health benefits, including antioxidant and antidiabetic properties. Based on different searches, this is the first scientific report with descriptive content on the potential use of M.olefiera leaves as an antioxidant and antidiabetic agent originated from Saudi Arabia.

Diabetes mellitus (DM) is a chronic disorder that affects lipid, carbohydrate, and protein functions, resulting in persisting hyperglycemia, as sourced from abnormal insulin secretion, insulin action, or both (Saltiel and Kahn, 2001). Potential phytocompounds affect multiple disease-related drugs with minimal adverse effects towards diabetic treatments (Nicolle et al., 2011). Phytocompounds sourced from different medicinal plants such as alkaloids, flavonoids, phenols, tannins, saponins, terpenoids, glycolipids, glycosides, anthocyanins and carotenoids was accounted as productive antidiabetic activity (Huang et al., 2016, Perera et al., 2016). Present studies have shown that the revelation from the phytochemical screening of MOLE showed saponin, flavonoid, gum, glycoside, tannin, phenol, starch and carbohydrate, where blood glucose lessens plant extract activities due to the presence of phytoconstituents. Other studies stated how polyphenols and flavonoids displayed reduced blood glucose, increased GLUT-2 expression in pancreatic beta cells, enhanced expression and promoting GLUT-4 translocation (Nisar et al., 2018).

Plant-derived phytoconstituents influenced metabolic glucose from the reduction of alpha-amylase activity, intensify insulin action and secretion, inhibits apoptosis, increased translocated and expressed glucose transporters, reduced gluconeogenesis, enhanced pancreatic beta-cell proliferation and protected pancreatic beta cells from oxidative stress and inflammation (Sayem et al., 2018). These phytocompounds mechanisms created a better understanding of designing antidiabetic drugs.

The linkage of pancreatic inflammation and oxidative stress assisted in diabetic progression and pathogenesis. Increased levels of free radicals produced from hyperglycemia-induced glucose autoxidation and protein glycosylation are crucial towards diabetic pathogenesis (Chen et al., 2015, Stirban et al., 2014). Pancreatic beta cells are sensitive to damages from nitric oxide and other free radicals (Hameed et al., 2015). Based on Gothai et al., 2016, Dal et al., 2016, some plant extracts demonstrated protective effects on pancreas beta-cells towards its antioxidant activities (Dal et al., 2016, Gothai et al., 2016). Also, MOLE contains antioxidant properties, as stated in the current study, it's suggested that protective effects on beta-cells correlate to antioxidant activities and affected antidiabetic activities. In addition, the inhibitory effect of phenolic compounds found within MOLE was assessed by the molecular docking. We run the molecular docking to identify the possible binding affinity and binding interactions of phenolic compounds in the active site of the human pancreatic α-amylase (HPA). The more negative docking scores is the more favorable binding affinity (Sabbah et al., 2010). The obtained interactions enhance the stabilizing of the complex and confirm the inhibitory effect of phenolic compounds found within MOLE for HPA.

## 5 Conclusion

The phytochemical and physiochemical findings of *Moringa oleifera* showed a potential natural drug. This study displayed the presences of natural phenols and flavonoids within *Moringa oleifera* ethanolic leaves extract that have favourable antioxidant effects counter to DPPH. Suggesting *Moringa oleifera* extract may be used to respond against the free radicals. The antidiabetic activity of the extract was studied on the alpha-amylase inhibitory effects. The results have created a credible mechanism towards the performance of leaf extract in *Moringa oleifera* due to inhibiting digestive enzymes. This is beneficial for diabetic patients to decrease or to evade diabetic-linked complications. So, comprehensive research should be done towards the isolation and differentiation of the active constituents of *Moringa oleifera* and exploring its medicinal bioactivity.

**Financial Support**: None.

**Author Contribution Statement**

All the authors enlisted are involved in project and drafted the article as well as provided a critical revision of the manuscript.

## CRediT authorship contribution statement

**Sridevi Chigurupati:** Project administration, Resources, Writing – review & editing. **Atheer Al-murikhy:** Methodology, writing – original draft. **Suliman A Almohammad:** Conceptualization, Data curation.**Yosif Almoshari:** Validation, Visualization. **Amira Saber Ahmad:** Formal analysis. **Shantini Vijayabalan:** Validation, Visualization, Writing – original draft. **Shatha Ghazi Felemban:** Funding acquisition, Investigation. **Vasanth Raj Palanimuthu:** Software, Writing – review & editing.

## Declaration of Competing Interest

The authors declare that they have no known competing financial interests or personal relationships that could have appeared to influence the work reported in this paper.
